# Epidemiology of Injury in Elite and Amateur Soccer Referees: A Systematic Review and Meta-analysis

**DOI:** 10.1007/s40279-025-02326-y

**Published:** 2025-09-29

**Authors:** Mohammad Alimoradi, Mohammad Alghosi, Mojtaba Iranmanesh, Mohammed Moinuddin, Nicola Relph

**Affiliations:** 1 Department of Sports Injuries and Corrective Exercises, Faculty of Sports Sciences, Shahid Bahonar University, Kerman, Iran; 2 Department of Sports Injury and Corrective Exercise, Faculty of Physical Education & Sport Sciences, University of Guilan, Rasht, Iran; 3 Department of Physical Education, Technical and Vocational University (TVU), Tehran, Iran; 4 School of Medicine and Dentistry, University of Lancashire, Preston, Lancashire PR1 2HE UK; 5 Faculty of Health, Social Work and Medicine, Edge Hill University, Ormskirk, Lancashire L39 4QP UK

## Abstract

**Background:**

The epidemiology of injury in soccer has traditionally focused on soccer players, rather than match officials. Although injury data on referees exist, no comprehensive review has summarized injury profiles in this population.

**Objective:**

To conduct a systematic review and meta-analysis of injury epidemiology in elite and amateur soccer referees, focusing on injury rates, types, locations, severity, and causes.

**Methods:**

PubMed (Medline), Web of Science, Scopus, CINAHL, and SPORTDiscus, covering their entire history up to 19 April 2025 were searched. This review included prospective and retrospective studies reporting injury incidence or prevalence among football match officials, with a study period of at least one season. Studies needed to specify injury definitions and include data on injury location, type, mechanism, or severity. Both male and female officials were eligible. Systematic reviews, commentaries, and letters were excluded. Study quality and risk of bias were evaluated using the STROBE-SIIS, in addition to the Newcastle–Ottawa Scale and funnel plots. Injury incidence rates were estimated using a random effects Poisson regression, accounting for heterogeneity and moderators. Heterogeneity was assessed with the *I*^*2*^ statistic.

**Results:**

A total of 17 studies were included, encompassing 3621 referees. The most frequent injuries were strains and sprains in the knee and ankle. The overall injury incidence was 2.19 injuries per 1000 h of exposure (95% CI 1.30–3.69). On-field referees experienced an incidence rate of 1.46 injuries per 1000 h of exposure (95% CI 0.76–2.81), while assistant referees had a lower rate of 0.84 per 1 h of exposure (95% CI 0.36–1.97). During matches, the injury incidence was 2.24 per 1000 h of exposure (95% CI 1.38–3.64), compared with 0.67 injuries per 1000 h of exposure during training sessions (95% CI 0.36–1.24). However, despite sensitivity analysis, there were still high levels of heterogeneity across included studies.

**Conclusions:**

Findings noted higher injury incidence during matches compared with training, and on-field referees compared with assistants. The variation in injury profiles highlights the importance of implementing targeted preventive strategies tailored to the unique demands of refereeing. However, there is still a lack of research in this population, especially in female referees.

**PROSPERO Registration Number:**

CRD42024497970.

**Supplementary Information:**

The online version contains supplementary material available at 10.1007/s40279-025-02326-y.

## Key Points

**Table:** 

On field referees have an estimated injury incidence rate of 1.46 injuries per 1000 h of exposure (95% CI 0.76–2.81), assistant referees have a lower rate of 0.84 per 1000 h of exposure (95% CI 0.36–1.97).
The injury incidence rate during matches was 2.24 per 1000 h of exposure (95% CI 1.38–3.64), whereas training sessions recorded a lower rate of 0.67 injuries per 1000 h of exposure (95% CI 0.36–1.24).
Injury surveillance measures were varied and there was a lack of evidence on female referees and overuse injuries.

## Introduction

Soccer is one of the most widely played sports globally, but it carries a substantial risk of injuries [1, 2]. According to the latest available official data (since no more recent published data are available) from Federation Internationale de Football Association (FIFA), approximately 840,000 on-field referees (OFRs) and assistant referees (ARs) participated in soccer in 2006 [3]. Physical fitness is a crucial aspect of the match officials’ performance as it allows them to stay close to the action and hence potential violations during matches [4]. Referees typically cover an average distance of approximately 11 km per match [5], maintaining a mean heart rate of 158.88 ± 3.99 bpm and during pivotal moments in the game, their heart rate can reach as high as 97% of their maximum [6]. Although OFRs cover 171% more high-intensity running distance than ARs, ARs perform 86% more accelerations [7]. Notably, a markedly elevated number of accelerations was associated with a substantially increased risk of non-contact injuries [relative risk (RR) = 5.11], whereas high acute high-speed distances also contributed to injury risk, albeit to a lesser degree (RR 2.55) [8]. Despite their differing physical demands, both OFRs and ARs require high levels of physical fitness [7, 9].

The OFR, commonly known as the 23rd player, along with the ARs, can experience considerable psychological and physical stress during a match, which can contribute to musculoskeletal injuries [10]. Indeed, research has suggested high-performance demands increase the risk of injury for match officials [11]. The incidence of injuries sustained by soccer referees may also be influenced by factors including age, the level of competition, location on the field, environmental conditions (such as surface and weather conditions), the site and timing of the injury, as well as sex [12–15]. In addition, soccer referees experience significant perceptual-cognitive demands, needing to quickly analyze visual cues and make decisions [5, 16] (often up to 200–250 foul or no-foul judgments per match [17]). Although skills such as pattern recognition and anticipation are essential, comprehension of the cognitive processes underlying these skills remains limited [5, 16].

In recent years, prospective and retrospective cohort studies have investigated injuries sustained by soccer referees [18–20], alongside several epidemiological studies documenting injury patterns over various seasons and tournaments [13, 20, 21]. Although a wealth of research exists on injuries among soccer referees [10, 18–23], there is a need for comprehensive studies that synthesize available epidemiological data on injury rates, types, locations, severity, and causes to identify unified patterns and inform targeted prevention strategies. A recent mini-review [24], focused solely on male soccer referees and including only five studies, limited the scope of data, and did not perform a meta-analysis, thus restricting the ability to quantitatively synthesize findings across different studies. It reported an injury incidence rate of 14.43 injuries per 1000 h of exposure during refereeing and 8.59 injuries per 1000 h of exposure during training. Furthermore, another recent systematic review [25] of five studies involving 433 head and 467 assistant referees identified various injury patterns in male soccer referees. Common post-match injuries included Achilles tendon, ankle, foot, and lower leg issues, while knee, hip, and groin injuries were frequent during physical tests. While this review provides valuable insights, our systematic review and meta-analysis aim to address these limitations by incorporating a broader range of studies. This comprehensive approach will enable a quantitative synthesis of injury prevalence, types, and anatomical locations, leading to more precise estimates of injury risks. Furthermore, it will offer deeper insights into injury patterns over referees’ careers, supporting the development of targeted prevention and risk reduction strategies. Such synthesis is essential for identifying common and severe injuries, understanding their specific locations, and pinpointing when these injuries are most likely to occur, whether during matches or training sessions [14].

Quantifying injury rates among soccer match officials is essential for developing targeted prevention strategies [19]. Without officials, competitive matches cannot take place. For this reason, we undertook a systematic review and meta-analysis to quantify the overall incidence of injuries among this population. Our secondary objective was to conduct sub-analyses to examine the overall incidence rate of injury for training and match play and to describe the nature, types, locations, and severity of injuries sustained by OFR and ARs.

## Methods

This study adhered to the Prisma in Exercise, Rehabilitation, Sport medicine and SporTs science (PERSiST) guideline [26, 27]. Moreover, this systematic review and meta-analysis was registered on the International Prospective Register of Systematic Reviews (PROSPERO) under the registration number CRD42024497970 on 9 January 2024.

### Search Strategy

A systematic search process was employed to identify potential studies. The search was performed across multiple databases, including PubMed/MEDLINE, Web of Science, Scopus, CINAHL, and SPORTDiscus, from their inception until 19 April 2025. The search strategy utilized Boolean operators to link the following keywords: soccer, football, injury, wound, incidence, prevalence, epidemiology, and referee. The detailed search history for each database is available in the Supplementary File 1. In addition, the reference lists of all included studies were manually scanned to identify any further eligible studies that may have been missed in the initial database search.

Initially, studies were imported into EndNote 20 (Clarivate, Philadelphia, PA, USA) for duplicate removal and then transferred to the Rayyan web application [28] for screening. The screening process was conducted by two authors (M.ALI. and M.ALG.) who were independently blinded to the other reviewer’s decisions and reviewed the titles and abstracts of the search results to identify relevant studies. Then, each potentially relevant study was evaluated independently by the same two blinded authors (M.ALI. and M.ALG.) based on the full text, making inclusion or exclusion decisions using the predefined criteria. Reasons for exclusion were recorded for any studies deemed irrelevant at this stage. In cases of disagreement during this review process, a third reviewer (N.R.) was consulted to make the final determination regarding the inclusion or exclusion of the study.

### Study Selection

Eligible studies had to meet the following criteria: (1) Prospective and/or retrospective study designs. (2) Reported the incidence or prevalence of injury and may have included one or more of the following epidemiological data formats: location of injuries; type of injuries; mechanism of injuries and severity of injuries of OFRs and ARs (injury incidence per 1000 h of exposure during training or matches, and/or injury prevalence reported with sufficient data in tables and figures for calculation) (3) Had a study period encompassing at least one season. (4) Included male and/or female match officials at any level. (5) Definition of injury provided. In addition, systematic / literature reviews, editorial commentaries, and letters to the editor were excluded from the analysis. Google Translate was utilized to interpret studies not published in English, ensuring that language barriers did not lead to the exclusion of relevant research.

### Data Extraction

A standardized data extraction form was developed a priori and used by two independent reviewers (M.ALI. and N.R.) who extracted data in a blinded manner to minimize bias. After initial independent extraction, the reviewers compared their results and resolved any discrepancies through discussion, involving a third reviewer (M.ALG.) if necessary. Extracted data encompassed: (1) total number and percentages of all injuries and/or number of match officials injured; (2) rate of injuries per 1000 h of exposure, or percentages if rates were not available; (3) sites and types of injuries by anatomical location per 1000 h of exposure, or percentages if rates were not available; (4) injury severity percentages and/or rates; and (5) total, training, and match exposure hours per on field referee and/or ARs. For each included study, information on study characteristics, including the study design, authors, publication year, and country of origin; characteristics of the study population, injury definition; and exposure, including the study period, number of participants, and seasons, were also extracted. If key variables such as injury count, exposure hours, or incidence rates were not reported, these were calculated from available data (e.g., exposure hours estimated as injury count divided by injury incidence multiplied by 1000). Minor rounding errors from these calculations were considered negligible [29]. To enhance comparability, injury definitions and severity classifications were aligned with established consensus statements where possible [29]. All extracted data were carefully checked for accuracy and consistency before analysis.

### Assessment of Reporting Quality and Risk of Bias

The reporting quality and risk of bias (ROB) of the included studies were evaluated using the the STROBE Extension for Sports Injury and Illness Surveillance (STROBE-SIIS) statement [30] and an adapted version of the Newcastle–Ottawa Scale (NOS) [31], respectively. The STROBE-SIIS provides guidance on the accurate reporting of observational studies concerning injury and illness in sports; however, it is not intended to serve as a direct assessment of study quality and has also been employed in previous studies [32, 33]. In particular, the STROBE-SIIS, which includes recommendations from the International Olympic Committee for observational studies in sports medicine [30], consists of 23 items spanning various categories. These categories include title and abstract, background/rationale, objectives, study design, setting, participants, variables, data sources/measurement, bias, study size, quantitative variables, statistical methods, descriptive data, outcome data, main results, other analyses, key results, limitations, interpretation, generalizability, funding, and ethics. To assess the ROB of the included studies, an adapted version of the NOS for cohort studies was employed. This choice was based on existing literature highlighting the NOS as an appropriate instrument for cohort studies [31]. The nine-item NOS evaluates three domains within studies: selection, comparability, and outcome. It is available in two versions, including one tailored for cross-sectional studies and another for cohort studies. Each domain provides a set of response options from which reviewers select the most appropriate for the study in question. Responses indicative of a low ROB are awarded a star, with a maximum score of 9 stars achievable per study. A greater number of stars indicates a lower ROB, representing higher methodological quality. In addition, studies with NOS star scores of 0–4 were classified as having a high ROB, scores of 5–6 as moderate risk, and scores of 7–9 as low ROB [31]. Using both the STROBE-SIIS and NOS standards concurrently has been demonstrated as good practice in prior systematic reviews [33, 34]. The assessment of reporting quality and ROB for each included study was conducted independently by two researchers (M.ALI. and M.ALG.). Any discrepancies between their evaluations were discussed, and disagreements were resolved through consensus with a third researcher (N.R.), who was involved when necessary.

### Statistical Analysis

The studies included in the analysis provided injury incidence rates per 1000 h of exposure. In cases where the specific rates were not reported, an attempt was made to calculate them using the available raw data. Similarly, if exposure hours were not provided, these were calculated from injury incidence rates and total injuries. If it was not possible to compute injuries and exposure times, the study was excluded from the meta-analysis.

The incidence calculation was performed using the formula:

$$\text{Incidence} = 1000 \times \left(\frac{\Sigma \text{injuries}}{\Sigma \text{exposure} \text{hours}}\right).$$

To estimate mean injury incidence rates with 95% confidence intervals, the data were analyzed using a random effects Poisson regression model, following the methodology described previously [35]. The response variable used was the number of observed injuries, adjusted (set as offset in the Poisson model) by the logarithm of the number of exposure hours. A random effects model was chosen as multiple rows of data from the same study were used. A weighting factor was applied, which considered the study exposure time (in hours) divided by the mean study exposure time (in hours). The main outcome variable was the count of injuries within a specific time duration for each study, and the count had an open upper limit. The specific variance estimator was the maximum likelihood estimator. Therefore, the outcome variable followed a Poisson distribution, which justified the use of a Poisson regression model [36]. This method has been previously applied to sports injuries in professional football [36].

The heterogeneity of the data was assessed using the *I*^2^ statistic, H-square (*H*^2^), Cochran’s *Q*-statistics (*Q*), and Tau-square (*τ*^2^). The *I*^2^ statistic quantifies the proportion of total variation across all studies that can be attributed to between-study heterogeneity. The *H*^2^ statistic describes the ratio of the observed variation and the expected variance due to sampling error. The *I*^2^ statistic quantifies the percentage of variability in the effect sizes that is not caused by sampling error. The *Q* statistic tests if the variation in a meta-analysis significantly exceeds the amount expected under the null hypothesis of no heterogeneity and also provides a *P* value. The *τ*^2^ gives the measure of between-study heterogeneity in the effect size. If *τ*^2^ is significantly greater than zero (0), study heterogeneity may be apparent.

A sensitivity analysis using the leave-one-out method was also conducted to explore if specific studies were causing the heterogeneity to be high. To examine potential publication bias, funnel plots and Egger’s test were used and interpreted. The model was estimated using a linear model on the estimated log incidence rate when the Egger’s statistical test was applied. This was done because the Poisson model for meta-analysis using the “metafor” package does not support the Egger’s test directly. Outliers were investigated by identification of extreme values that fell outside the three-times median absolute deviation (MAD) from the median limit [37] and visually using a boxplot of incidence values.

To examine the potential impact of high heterogeneity on the overall model, moderator analyses of injury per 1000 h were conducted. Moderator variables included age, referee role (on field referee/AR/both), level of referee (elite/semi-professional/amateur), injury setting (match/training/both), study design (prospective/retrospective), exposure time (12 months, 3-seasons, career, last match, one competition), and age of study. All statistical analyses were conducted using R, version 4.4.3 [38].

### EDI Statement

The search strategy aimed to capture any group of referees; we did not exclude on the basis of characteristics. The author team is an international mix of male and female academics from multiple disciplines.

## Results

### Search Results

**Fig. 1:**
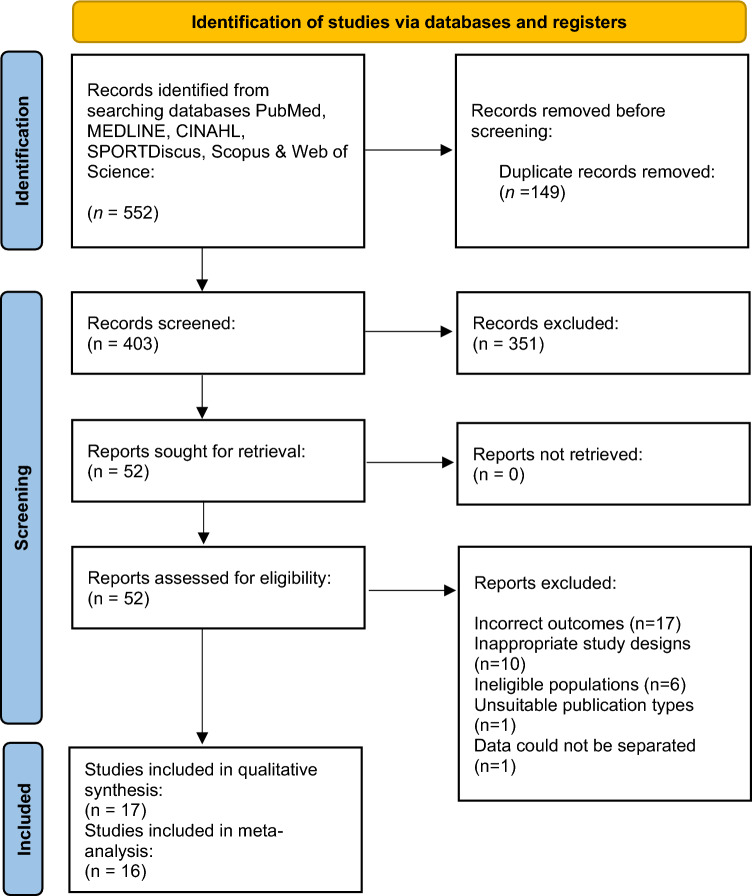
PRISMA 2020 flow diagram for new systematic reviews, including searches of databases and registers

In total, we found 552 titles from six databases (see Fig. 1). During initial screening, 149 studies were removed due to being duplicates (27.0%). Following the title and abstract review, a further 351 studies (63.6%) were deemed irrelevant and removed. Hence, full-text screening was completed on 52 titles (9.4%). After completing this process, 35 studies were excluded as they did not meet the inclusion criteria; specifically, 17 did not collect measures of injury (incorrect outcomes), 10 were not retrospective or prospective design (inappropriate study designs), 6 did not include match officials (ineligible populations), 1 was an editorial (unsuitable publication types), and 1 did not provide separate data for OFRs and ARs. Therefore, 17 studies were included in the systematic review and 16 studies in the meta-analysis.

### Descriptive Characteristics of the Included Studies

The characteristics of the 17 included studies are presented in Table 1. The studies, which were published between 2009 and 2022, included 3621 soccer referees (mean ± SD age: 33.03 years ± 3.5). The studies were conducted in various locations, including Europe (*n* = 8), Asia (*n* = 4), and South America (*n* = 5). A total of 2801 OFRs and 820 ARs, of whom 3460 (95.56%) were male and 161 (4.44%) were female, were included in the studies. The level of play varied across studies, with studies including professional referees [10, 12, 14, 15, 18–23, 39–41], semi-professional referees [14, 15, 42], amateur referees [10, 14, 15, 19, 41, 43], junior referees [15], and achilles referees [41]. Of the included studies, five studies had a prospective design [10, 18, 21, 23, 39], seven studies had a retrospective design [14, 19, 22, 40–42, 44], and two studies implemented a retrospective and prospective design [12, 13]. One study used a prospective cross-sectional design [20] and one randomized controlled trial’s control group was included as it had a prospective design [43]. The minimum, maximum, and average length of the studies for 14 included papers in the current study were 20 days [12], 3 years [20], and 1 year, respectively. In addition, three studies included data from the entire career of referees [22, 40, 41].

**Table 1:** Characteristics of included studies

Study	Country	Study design	Length/setting	Sample size, sex distribution	Age (years)	Level	Level of play detail
Bizzini et al., 2009 [12]	China (Pre-World Cup camps)	Retrospective and prospective	One competition/Women's World cup 2007	81, F	35.0 ± 4.4	Elite	Soccer referees (OFRs and ARs) preselected and selected for the FIFA Women’s World Cup 2007
Bizzini et al., 2009 [15]	Switzerland	Retrospective	One season/professional and amateur soccer	489 (481 M, 8 F)	36.6 ± 14.9	Amateur	Referees from professional and semi-professional (super and challenge leagues), high-level amateur (first and second leagues), low-level amateur (third–fifth leagues), junior (all leagues with players up to 20 years of age)
Bizzini et al., 2009 [44]	Switzerland	Retrospective cohort	One season/professional soccer	71 (66 M, 5 F)	M (36.0 ± 5.3), F (31.0 ± 6.0)	Amateur	All referees (OFRs and ARs) officiating in the Swiss Super and Challenge League (first and second national divisions)
Bizzini et al., 2009 [13]	Germany (pre-world cup camps)	Retrospective survey and prospective study	One competition/Men's World cup 2006	123, M	41.0 ± 3.7	Elite	Referees (OFRs and ARs) preselected and selected for the 2006 FIFA Men’s World Cup
Wilson et al., 2011 [39]	Ireland	Prospective cohort	One season/amateur soccer	31 (27 M, 4 F)	33.2 ± 6.2	Elite	Elite referees (OFRs and ARs)
Paes et al., 2011 [42]	Brazil	Retrospective survey	One season/Paraná’s Football Championship	200, M	32.0 ± 6.0	Semi-professional	Semi-professional; soccer referees (OFRs and ARs) officiating in the Paraná’s Football Championship (First and Second Divisions)
Gabrilo et al., 2013 [22]	Croatia	Retrospective	Whole career/professional soccer	342, M	32.9 ± 5.02	Elite	Referees (OFRs and ARs) had various competitive levels—international and national level
Kordi et al., 2013 [18]	Iran	Prospective	One season/professional soccer	74, M	OFRs = 37.30 ± 3.20; ARs = 36.30 ± 4.30	Elite	Referees (OFRs and ARs) selected who officiated in the 2009–2010 season of the Iranian Premier Football League
Da Silva et al., 2014 [41]	Brazil	Retrospective	Whole career/professional and amateur soccer	101, M	29 ± 7.55	Mixed—unable to separate data	Referees (OFRs and ARs) (professional, amateur and leisure) who were accredited by the Santa Catarina Soccer Federation (FCF)
De Oliveira et al., 2016 [40]	Brazil	Retrospective	Whole career/professional soccer	36, M	32.0 ± 5.6	Elite	Referees were from FPF and FGF that act at the A série of the Brazilian championship
Boneti Moreira et al., 2017 [23]	Brazil	Prospective	One season/professional soccer	220, M	29.31 ± 6.65	Semi-professional	Referees included that registered with the Paraná Football Federation and participate annually in national and state championships
Vieira et al., 2019 [20]	Brazil	Prospective cross-sectional	Three seasons/professional soccer	257, M	32.9 ± 5.0	Elite	Professional soccer referees who registered in Federação Paulista de Futebol (FPF)
Matute-Llorente et al., 2020 [19]	Spain	Retrospective	One season/professional soccer	232, M	Elite referees: (1st league: OFRs = 38.9 ± 3.0, ARs = 36.3 ± 5.5; 2nd league: OFRs = 34.5 ± 3.4, ARs = 30.8 ± 4.9). Non-elite referees: (2ndB league: OFRs = 30.2 ± 4.3	Elite and amateur	Referees (OFRs and ARs) were elite: Spanish La Liga Santander (1st division), and La Liga Smart Bank (2nd division) leagues. Nonelite: Spanish 2nd B league
Al Attar et al., 2021 [43]	Saudi Arabia	Randomised controlled trial (control group data only)	One season/amateur soccer	100, M	31.6 ± 4.1	Amateur	Amateur soccer referees
Szymski et al., 2021 [14]	Germany	Retrospective cohort	One season/professional and amateur soccer	923 (796 M, 127 F)	Professional (33.9 ± 11.0), semi-professional (31.7 ± 12.3) and amateur (35.1 ± 17.4)	Elite, semi-professional and amateur	Professional, semi-professional, and amateur referees (OFRs and ARs)
Moen et al., 2022 [21]	Norway	Prospective cohort	One season/professional soccer	55 (38 M, 17 F)	31 [20–53]^a^	Elite	Norwegian top-level football referees (OFRs and ARs) (the highest national level and FIFA-referees)
Senisik et al., 2022 [10]	Turkey	Prospective cohort	One season/professional soccer	286, M	24.9 ± 6.1	Mixed – unable to separate data	Professional and amateur referees (OFRs and ARs). No referees were full-time professionals

M, male; F, female; OFRs, on-field referees; ARs, assistant referees

^a^Reported as median (range)

In terms of injury definitions, included studies employed a variety of definitions, including time loss injuries [19, 40–42], time injuries resulting in a referee leaving the field [10, 12–15, 18–20, 22, 39, 43, 44], any physical complaint resulting from refereeing [19], and injuries requiring medical attention [21]. Among the included studies, only one paper did not provide a formal injury definition; instead, it employed an operational definition to categorize and specify injuries consistently across the study [23]. This study employed the Inquérito de Morbidade Referida to assess musculoskeletal injuries during 8 months. This instrument includes detailed criteria for identifying injury events (such as type, cause, mechanism, and consequences) aligning with our inclusion criteria. The units of measurement used for incidence were also provided in 16 studies, with some reporting incidence per 1000 referee hours [12, 13, 15, 21, 22, 44] and per 1000 referee-exposures [10, 14, 18–20, 39–43]. The anatomical location of injuries was provided in 17 studies, while data on injury type and severity were reported in 17 and 15 studies, respectively.

### Injury Incidence

**Fig. 2:**
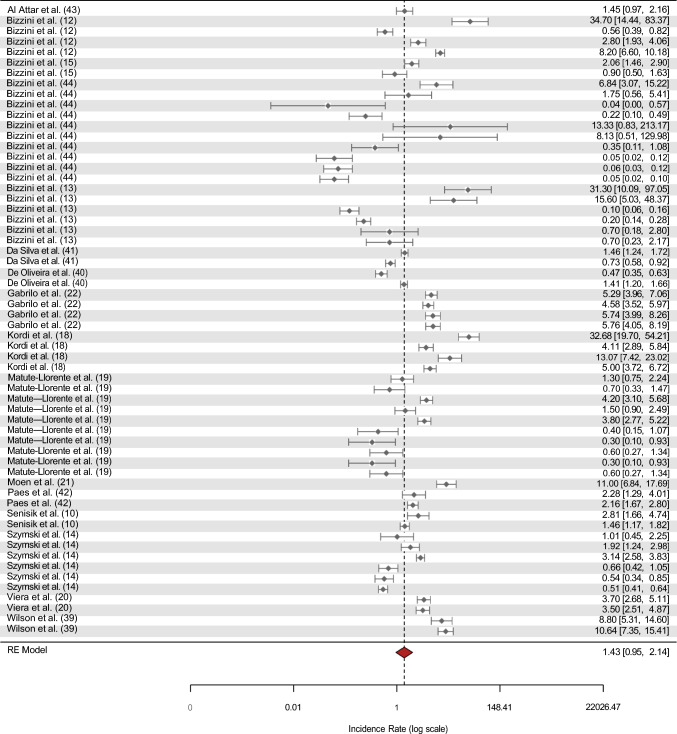
Forest plot for overall injury incidence, plotted as injury incidence and 95% CIs. Some studies are included multiple times, which is due to the reporting of injuries in sub-groups, such as sex or match versus training settings

In the 17 included studies, 2195 injuries were reported. The results of the random effect models for injury incidence showed an overall incidence of 1.43 injuries per 1000 h of exposure (95% CI 0.95–2.14, *I*^2^ = 98.46%) for all referees. Overall injury incidence is displayed in Fig. 2.

### Sensitivity Analysis, Publication Bias, and Outlier Analysis

The leave-one-out analysis indicated that the exclusion of no specific study improved the heterogeneity or effect estimates. Funnel plots from both original and log incidence rate models indicated no evidence of publication bias. Further, the Egger’s test suggested that there was no significant publication bias present in the included studies (*z* = − 0.9838, *P* = 0.3252). No outliers were identified in the included studies. See Supplementary File 2 for details of these analyses.

### Moderator Analysis

Because of the high heterogeneity measure in overall injury incidence, moderator analyses provided more information (see Table 2), all RRs are presented as per 1000 h of exposure. Several factors were considered for moderator analysis. Age (RR 1.002, 95% CI 0.909–1.105, *P* = 0.962) and referee level (elite reference, amateur RR 1.330, 95% CI 0.585–3.023, *P* = 0.496; semi-professional RR 0.939, 95% CI 0.456–1.933, *P* = 0.864) did not significantly moderate the model.

**Table 2:** Risk ratio per 1000 h of exposure and moderator variables of injuries in soccer referees

Moderator variables	Category	*N* (%)	RR	*P* value	95% CI (lower)	95% CI (upper)
Age	Median (interquartile range)	33.6 (32.7, 36.6)	1.002	0.962	0.909	1.105
Sex	Male	35 (58%)	Ref			
	Female	4 (6.7%)	84.133	0.000*	8.593	823.771
	Unspecified	21 (35%)	1.138	0.702	0.588	2.203
Referee role	Referee	26 (43%)	Ref			
	Assistant referee	17 (28%)	0.671	0.103	0.416	1.083
	Both	17 (28%)	0.030	0.000*	0.006	0.153
Match type	Match	38 (63%)	Ref			
	Training	21 (35%)	0.379	0.000*	0.250	0.574
	Both	1 (1.7%)	0.070	0.009*	0.010	0.510
Referee level	Elite	35 (58%)	Ref			
	Amateur	17 (28%)	1.330	0.496	0.585	3.023
	Semi-professional	4 (6.7%)	0.939	0.864	0.456	1.933
	Mixed level	4 (6.7%)	0.810	0.571	0.390	1.682
Injury definition	Time loss	28 (47%)	Ref			
	Referee leaves field	21 (35%)	0.108	0.000*	0.032	0.361
	Medical attention	11 (18%)	0.295	0.000*	0.156	0.556
Study design	Prospective	15 (25%)	Ref			
	Retrospective	45 (75%)	0.249	0.000*	0.131	0.477
Exposure time	12 months	38 (63%)	Ref			
	3-sessions	2 (3.3%)	0.054	0.000*	0.013	0.226
	Career	11 (18%)	0.137	0.000*	0.069	0.273
	Last match	5 (8.3%)	1.809	0.203	0.727	4.505
	One competition	4 (6.7%)	3.498	0.044*	1.032	11.851
Age of study	Median (interquartile range)	10.5 (4.0, 15.0)	0.736	0.001	0.617	0.879

*Indicates a statistically significant moderator at 5% level of significance

Sex was a mediating factor; females (RR 84.133, 95% CI 8.593–823.771, *P* < 0.001) had a significantly higher risk of injury than males. Referee role was also a mediating factor; studies that combined OFR and AR data had a significantly lower risk of injury (RR 0.030, 95% CI 0.006–0.153, *P* < 0.001) compared with OFRs. OFRs and ARs did not differ in risk of injury. Training settings also offered a significantly lower risk of injury (RR 0.379, 95% CI 0.250–0.574, *P* < 0.001) when compared with match settings, as did studies that combined match and training setting (RR 0.379, 95% CI 0.250–0.574, *P* < 0.001).

Injury definitions that included the referee leaving the field (RR 0.108, 95% CI 0.032–0.361, *P* < 0.001) or requiring medical attention (RR 0.295, 95% CI 0.156–0.556, *P* < 0.001) recorded significantly lower risk of injury compared with time loss injury definitions. Recording injury data on a referee’s whole career or 3 months significantly reduced the RR of injury when compared with one season of data collection (RR 0.223, 95% CI 0.118–0.423, *P* < 0.001 and RR 0.054, 95% CI 0.013–0.226, *P* < 0.001, respectively), as did a retrospective study design when compared with prospective study designs (RR 0.249, 95% CI 0.131–0.477, *P* < 0.001). However, there was an increased risk of injury in one competition (e.g. the World Cup) compared with 12-months (RR 3.498, 95% CI 1.032–11.851, *P* = 0.044). The age of the study was also a moderating factor; the older the study, the lower the risk of injury (RR 0.736, 95% CI 0.617–0.879, *P* = 0.001).

### Injury Characteristics

All included studies provided information on the distribution of the anatomical location of referee injuries [10, 12–15, 18–23, 39–44] (Table 3). A total of 12 studies investigated the location of injuries in both referees; OFRs and ARs [10, 12–14, 18, 19, 21, 22, 39, 41, 42, 44]. Among the studies included, 2195 injuries reported that calf/lower leg (*n* = 691; 31.48%), thigh (*n* = 556; 25.33%), and knee (*n* = 461; 21%), were considered as commonly injured areas of the body in soccer referees. A total of 17 studies provided information on the type of injuries (Table 2) that referees sustained [10, 12–15, 18, 19, 21–23, 39–44]. Exactly 11 studies reported either strains (*n* = 507; 52.48%) or sprains (*n* = 205; 21.22%) as the common injury types for soccer referees [12, 13, 15, 18, 19, 22, 23, 40–42, 44]. Moreover, five studies categorized the injury types as acute/trauma (*n* = 200; 47.85%) or chronic/overuse (*n* = 215; 52.15%) [10, 18, 21, 39, 43]. While the injury mechanism was provided in one study [14], other studies reported details of the gradual onset of injuries [21] and details of the sustained injuries [12, 44]. Szymski et al. [14] reported that soccer referees sustained 31 injuries, accounting for 13.90% as contact injuries, and 192 injuries, accounting for 86.10% as noncontact injuries. A total of 13 studies presented information regarding the severity of the documented injuries (Table 2) [12–15, 19–23, 39, 43, 44]. It is important to note that none of the studies included in the analysis reported any injuries of a catastrophic or life-threatening nature.

**Table 3:** Injury details for soccer referees from the included studies

	Count	Percentage	Sample size
Location			
Groin/hip	92	4.19	3037
Thigh	556	25.33	3409
Knee	461	21.00	3590
Ankle/foot/toe	295	13.43	3290
Calf/lower leg	691	31.48	3621
Heel/plantar fascia	3	0.13	1035
Lower back/trunk/abdomen/pelvic/sacrum	71	3.23	2707
Upper extremity/rib/clavicle	19	0.86	2074
Head/face/neck	7	0.35	2444
Total	2195	100	
Type			
Strain	507	52.48	2286
Sprain	205	21.22	2255
Initial	24	2.48	100
Recurrent	9	0.93	386
Contusion/hematoma/stenosis	8	0.83	1055
Lesion	37	3.83	1025
Tendon injury	59	6.11	1513
Concussion/dizziness	5	0.52	831
Fracture/dislocation	32	3.31	1400
Spasm/pain/nonspecific pain	44	4.55	919
Tendinitis/bursitis	32	3.31	860
Lumbago	2	0.21	232
Wound/laceration	2	0.21	465
Total	966	100	
Acute/chronic			
Acute	200	47.85	1412
Chronic	218	52.15	1054
Total	418	100	
Cause of injury			
Contact	31	13.90	923
Non-contact	192	86.10	1054
Total	223	100	

### Estimated Injury Incidence

Injury incidence was estimated using all available data from the included studies (see Table 4). Females had an estimated injury incidence rate of 4.27 (95% CI 1.02–17.79) per 1000 h of exposure compared with male’s incidence rate of 1.79 (95% CI 1.15–2.76) per 1000 h of exposure. Meta-analysis for role of the referee subgroup further revealed that OFRs have an estimated injury incidence rate of 1.46 injuries per 1000 h of exposure (95% CI 0.76–2.81), ARs have a rate of 0.84 per 1000 h of exposure (95% CI 0.36–1.97), and when both were considered together, the estimated incidence rate was 2.19 per 1000 h of exposure (95% CI 1.30–3.69). The meta-analysis also indicated that during match exposure, the estimated injury incidence rate was 2.24 per 1000 h of exposure (95% CI 1.38–3.64), whereas training sessions recorded a lower rate of 0.67 injuries per 1000 h of exposure (95% CI 0.36–1.24). Further, the results indicated that in prospective studies, the estimated incidence rate of injuries was 6.65 per 1000 h of exposure (95% CI 4.03–10.98), while retrospective studies reported a lower rate of 0.84 injuries per 1000 h of exposure (95% CI 0.55–1.27). However, there were high levels of heterogeneity in all estimations (*I*^*2*^ range from 95.25 to 98.59).

**Table 4:** Estimated incidence rate of injuries in soccer referees per 1000 h of exposure and heterogeneity measures in different subgroups

	Variable	Incidence (95% CI)	Tau-square (${\tau }^{2}$)	*I*-square (${I}^{2}$)	*H*-square (${H}^{2}$)
Sex	Male	1.785 (1.153, 2.764)	1.635	98.073	51.9
	Female	4.266 (1.023, 17.791)	2.037	98.273	57.9
	Unspecified	0.732 (0.321, 1.669)	3.233	98.169	54.628
Referee role	Referee	1.464 (0.763, 2.813)	2.644	98.593	71.077
	Assistant referee	0.837 (0.356, 1.967)	2.908	97.937	48.462
	Both	2.188 (1.297, 3.691)	1.148	97.528	40.459
Match type	Match	2.242 (1.381, 3.639)	2.1	98.089	52.325
	Training	0.667 (0.358, 1.244)	2.008	98.347	60.482
	Both	na	na	na	na
Injury definition	Time loss	0.809 (0.396, 1.649)	3.284	98.806	83.785
	Referee leaves field	3.179 (2.04, 4.953)	1.03	97.235	36.161
	Medical attention	1.121 (0.56, 2.242)	1.234	94.033	16.759
Study design	Prospective	6.648 (4.027, 10.976)	0.869	95.254	21.068
	Retrospective	0.837 (0.553, 1.269)	1.817	98.05	51.294
Exposure time	12 months	1.698 (1.124, 2.565)	1.57	97.296	36.984
	3-sessions^a^	3.6 (2.858, 4.535)	0	0	1
	Career	0.251 (0.116, 0.542)	1.561	98.446	64.353
	Last match	6.699 (5.32, 8.435)	0.013	21.113	1.268
	One competition	10.418 (2.754, 39.408)	1.565	90.032	10.032

Na, one study only

^a^Estimates based on two studies only

### Reporting Quality and Risk of Bias of the Included Studies

The overall reporting quality, measured using the 23-item STROBE-SIIS checklist, was reflected in a mean ± SD score of 18.00 ± 2.74, ranging from 12 to 22. The mean ± SD ROB, evaluated with the nine-item NOS, was 7.29 ± 0.45, with a range of 7–8. All included studies received a NOS score of ≥ 7, indicating a low ROB. Detailed individual ratings for both the NOS and STROBE-SIIS assessments are available in Supplementary Files 3 and 4, respectively.

## Discussion

Establishing the extent of an injury problem, including both incidence and severity, in a sport is the first step toward injury prevention [45, 46]. This study is the first systematic review and meta-analysis focused on the injury characteristics of soccer referees, aiming to quantify the incidence and epidemiological data of injuries within this group. Furthermore, it analyzed variations in incidence across different subgroups, including referee roles, referee level, injury setting, and study designs. Notably, there was a significant paucity of data concerning female referees, with only 6 out of 17 included studies [12, 14, 15, 21, 39, 44] reporting on women’s injury data, and just a single study [12] specifically addressing female referees. The study also examined the relative injury risk per 1000 h in relation to factors such as age, sex, level of refereeing, exposure time, and study context across diverse settings.

### Overall Injury Incidence and Estimated Injury Incidence

The meta-analysis revealed that soccer referees have a significantly lower injury incidence rate of 1.43 injuries per 1000 h of exposure compared with soccer players, who report rates ranging from 5.5 to 7.9 injuries per 1000 h of exposure depending on age and sex [34, 47]. This difference may be attributed to the lower intensity of effort required by referees, whose roles involve prolonged moderate-intensity running rather than the short, explosive actions typical of players [5, 9, 48–50], but also to a fundamental distinction since referees do not play the ball and thus their risk of contact injuries, which are common among players, is essentially zero. However, it is important to consider other key factors that may influence these findings. Variations in injury reporting systems across studies can affect injury incidence estimates, potentially leading to inconsistencies when comparing referees and players [51, 52]. For example, the injury rates among referees may have been underestimated due to less rigorous medical follow-up and injury surveillance compared with players, who often have dedicated medical teams and systematic reporting protocols [20, 53]. Furthermore, the older average age of referees—about 15 years older than players [21, 54]—may have contributed to differences in injury patterns and risks, as well as their typically longer careers, which increase exposure to both acute and overuse injuries [21]. Taken together, these factors suggest that while the lower injury incidence in referees reflects differences in physical demands, methodological and contextual considerations must also be acknowledged when interpreting these comparisons.

Referees experience a higher injury incidence during matches, with a rate of 2.24 per 1000 h of exposure, compared with 0.67 per 1000 h of exposure during training. The injury risk in training was also significantly lower (RR 0.379, 95% CI 0.250–0.574) than matches. This increased risk during matches likely stems from a combination of physiological, biomechanical, and psychological factors. The intense physical demands [55], such as covering 10,000 to 13,000 m and performing rapid directional changes, place significant biomechanical stress on the lower limbs [56]. At the same time, psychological pressure from high-level scrutiny and mental fatigue during matches contribute to the risk by impairing focus and decision-making [55, 57, 58]. These factors together explain the higher injury prevalence observed in match situations.

Moreover, the potentially amateur status of referees globally, contrasted with the professional environment of players, may influence their physical and psychological conditions. This disparity in experience and age highlights the need for further research to explore the factors influencing these differences in injury rates and to develop targeted prevention strategies for both referees and players. The current study included a total of 3621 referees, with 2110 professional referees and 1511 amateur referees. The amateur referees were notably younger and had less experience compared with the professional players in the study. This disparity in age and experience between referees and players may have influenced the higher injury rates observed among amateur referees.

### Moderators: Age and Sex

The analysis of RRs in our study on the epidemiology of injuries among soccer referees indicated that age of referee did not have a significant impact on injury outcomes. Specifically, the RR for age was 1.002, with a 95% CI ranging from 0.909 to 1.105 and a *P* value of 0.962, suggesting that age variations did not meaningfully affect injury risk among referees. However, it is important to note that this finding likely reflects the fact that nearly all included studies focused primarily on acute injuries. Overuse injuries, which may accumulate with longer careers and aging, are often underreported owing to limitations in injury documentation methodologies. Given the typical length of referees’ careers, it can be speculated that the burden of overuse injuries may increase with age [59], but further specific research is needed to clarify this.

In contrast, female referees had a significantly higher injury RR compared with male referees and an estimated injury incidence over two times higher than males. Female referees cover an average of 9.5 km per match, with approximately a quarter of this at speeds greater than 13 km/h [60]. However, some research suggests female referees fatigue in the second half of matches [60, 61], although this has been contested [62]. If fatigue is apparent, this could increase the risk of injury and may be attributed to the relatively recent professionalization of the women’s game, with female referees potentially not having access to appropriate training and injury prevention programs at the same level as male referees. Indeed, research suggests that female referees require more targeted strength and power training to improve fitness [63]. It is also established that females are more at risk of specific musculoskeletal injuries such as anterior cruciate ligament damage [64]. However, as previously stated, there is a lack of research available on female referees and hence this population should be prioritized in future epidemiological research.

### Moderators: Referee Role and Level

In examining the characteristics of injuries sustained by OFRs compared with ARs, significant differences emerge that warrant further investigation. The incidence of injuries among ARs is notably lower, reported at 0.84 injuries per 1000 h of exposure, in contrast to 1.46 injuries per 1000 h of exposure for OFRs. This discrepancy indicates that OFRs face a 54% higher risk of injury, being 1.54 times more likely to sustain an injury than their AR counterparts. Such differences can be attributed to the distinct roles and activities each referee undertakes during a match. OFRs typically engage in short, intense sprints interspersed with longer periods of low-intensity activity [65], while ARs often sprint at high intensities over longer distances, adapting to the dynamic nature of the game [49]. Supporting this, the distribution of movement types also varies significantly between the two officiating roles. OFRs primarily utilize forward running to maintain visual contact with the ball and players, which involves more explosive, short-duration efforts. Conversely, ARs spend a considerable amount of their time performing sideways movements, accounting for approximately 30% of their total distance covered [66, 67], which are less intense but require sustained lateral activity. In addition, ARs tend to remain stationary about 57% of the time and walk during 24%, with only about 1.4% of their activity dedicated to sprinting [67]. Furthermore, training for OFRs should focus on maintaining high-intensity running throughout matches, especially in the second half when their performance declines, with high-intensity interval training that simulates match conditions and improves acceleration and recovery [67]. In contrast, ARs need specialized drills targeting their movement patterns, including frequent lateral movements and short sprints. These drills should aim to enhance repeated sprints and sideways agility without sacrificing positional awareness, while also addressing the decline in their effective index during the second half [67]. These activity patterns highlight how the differing movement dynamics contribute to injury risk variations between the roles.

Moreover, some studies included in the analysis [12, 13, 18, 22, 44] revealed that ARs experience a higher frequency of trunk and upper extremity injuries compared with OFRs. This disparity emphasizes the necessity for injury prevention strategies that are specifically designed to address the unique challenges faced by each referee role. Currently, the FIFA 11 + Referees Program includes exercises for both ORs and ARs across its three parts [43, 68–70]. In this regard, Al Attar et al., in a randomized controlled trial study (Level 1 evidence), reported that this program reduced injuries by 65% in male amateur referees [43]. The program’s parts 1 and 3 serve as quick, practical warm-ups that improve movement quality and short-term performance [70]. In addition, the program enhances change-of-direction maneuverability, which is crucial for injury prevention [71]. Moreover, evidence from Weston et al. reports injury reduction in elite referees during the 2006 and 2010 World Cup tournaments, highlighting the program’s impact beyond amateur levels [5]. These findings highlight the potential for targeted neuromuscular training to further protect ARs, suggesting that integrating specific injury prevention strategies could support referees’ safety and performance more effectively. Further research is needed to assess its long-term benefits and broader applicability.

Interestingly, the level of referee grouped as elite, semi-professional, and amateur did not significantly moderate injury incidence, and these data suggest all levels of referee are at equal risk of injury. This finding may conflict with the current understanding of professionalism in refereeing, for example amateur referees, who often operate under less structured physical and psychological preparation regimes, and with limited medical resources, may experience more injuries and be less likely to report injuries accurately [14]. Conversely, it could be thought that elite referees, such as those in UEFA competitions, benefit from supervised training and regular medical help, leading to more comprehensive injury documentation [20, 22]. The results of our study do not support these findings; however, it is important to note that only two of the included studies compared different levels of refereeing, with only one paper comparing all three levels of refereeing [14]. Therefore, future research should include comparisons of all three levels of refereeing and detailed measurement of training characteristics to explore if this negates the potential impact of professionalism.

### Moderators: Injury Setting

Our meta-analysis underscores that injury incidence among soccer referees varies significantly by setting, with a markedly significant difference in RR and a markedly higher rate during matches (2.24 injuries per 1000 h of exposure) compared with training sessions (0.67 injuries per 1000 h of exposure). Similar findings were reported by Rodríguez et al. [24], who documented 14.43 injuries per 1000 h of exposure during matches versus 8.59 during training, suggesting that the physical environment of matches inherently carries greater injury risk owing to increased physical demands and unpredictability. The comparatively low injury rate could be an underestimate caused by underreporting.

Several factors point to the likelihood of underreporting of training injuries. First, the lack of dedicated medical support for referees, unlike players with access to medical teams, means that minor or moderate injuries during training often go undiagnosed and unreported [53]. Furthermore, the current data collection methodologies influence injury reporting. Prospective studies tend to report higher injury rates (up to 20 injuries per 1000 h of exposure) compared with retrospective studies, where recall bias causes underreporting [22, 72]. This suggests that retrospective studies may underestimate training injuries because referees forget or fail to report minor incidents. Moreover, the research focus often emphasizes match injuries, creating a knowledge gap concerning training injuries [20].

In addition, inconsistencies in injury definitions do not align well with refereeing realities, where injuries may not result in medical care or extended absence but still impact performance. Indeed, included studies in our analysis reported injury definitions as a “referee leaving the field” “referee requiring medical attention,” and time-loss injury definitions, which are all considerably different measures of injury. A referee would not need to leave the field if the injury occurred during training. The analysis supported the variability in definitions as injury definition was a significant moderator with the “referee leaving the field” producing the highest injury incidence (3.18 injuries per 1000 h of exposure) compared with the lowest “time-loss” incidence (0.809 injuries per 1000 h of exposure).

Considering these factors, it is plausible that the low number of reported training injuries reflects a combination of genuinely lower risk exposure during training and significant underreporting. The structural and methodological barriers likely result in an underestimation, emphasizing the need for enhanced reporting systems and targeted research to accurately assess training injury risks among referees.

### Moderators: Study Design

Recording injury data over a referee’s entire career indicates a significant reduction in the RR of injury, with a RR of 0.137 (95% CI: 0.069–0.273, *P* < 0.001), compared with data collected over a single season. Retrospective studies [14, 19, 22, 40–42, 44] also showed lower RR (RR of 0.249, 95% CI 0.131–0.477, *P* < 0.001) than prospective designs [10, 18, 21, 23, 39]. Combining data from retrospective studies (which analyze injury data from various time points or past records) with prospective studies (which monitor injuries during specific events such as tournaments or over multiple seasons) presents particular methodological challenges. Retrospective analyses may be prone to recall bias [72], incomplete records, and inconsistencies in injury reporting, all of which can affect data accuracy. While prospective studies generally reduce some of these risks through systematic data collection, it remains essential to standardize injury definitions and categorization, particularly distinguishing injuries among assistant referees and other officials. To improve data validity and deepen understanding of injury patterns among soccer referees, adopting prospective study designs with clear, systematic injury definitions is highly recommended. It is also recommended that future research adopts prospective monitoring protocols with real-time data recording to minimize recall bias.

Conversely, risk of injury increased during one condensed competition, such as the World Cup (RR 3.498 95% CI 1.032–11.851, *P* = 0.044) compared with one season. This is likely owing to the shorter periods of recovery in tournaments. Therefore, it is recommended that injury prevention strategies are designed for, tested, and evaluated during tournaments and not just throughout a soccer season.

### Injury Characteristics

In soccer referees, injuries to the calf, lower leg, thigh, and knee, including strains and sprains, were commonly observed. These injuries may be influenced by multiple factors, such as deficits in physical preparation [22], cumulative overload [20, 55], and movement biomechanics [56]. Referees are required to maintain high levels of agility and endurance while performing rapid directional changes [71], which can place significant stress on the lower extremities. Insufficient physical conditioning or inadequate warm-up routines might predispose referees to muscle strains, especially in the calf and thigh regions, by reducing muscle resilience. The repetitive nature of sprinting, abrupt stops, and lateral movements can lead to cumulative overload, resulting in muscle fatigue and overuse injuries [73]. In addition, biomechanical factors such as improper running techniques or imbalance may contribute to strains and joint injuries, notably in the knee [74]. The combination of these elements, along with running on varied surfaces and the physical demands of officiating, underscores the importance of comprehensive preparation and biomechanical assessments to prevent injury risks.

The observation that injuries among soccer referees were roughly evenly split between acute/trauma and overuse types was intriguing and highlights the multifaceted nature of injury risk in this population. However, it is important to recognize that injury patterns may differ significantly between roles, such as ARs and ORs, owing to their distinct movement patterns and physical demands. ARs, for example, perform more lateral shuffling and rapid directional changes, which may lead to a different injury profile compared with ORs, who typically engage in more linear running and positioning [67]. Furthermore, the cumulative effects of repetitive motions and prolonged physical exertion, especially over an entire career, play a crucial role in injury development. Longer career lengths can increase exposure to overuse injuries or gradual-onset injuries [21], emphasizing the importance of implementing targeted injury prevention and management strategies across all levels. This approach should include tailored training, recovery protocols, nutrition, and other preventive measures, recognizing the unique demands and injury risks associated with each referee role.

### Strengths and Limitations

The strengths of this systematic review and meta-analysis lie in its adherence to the PERSiST guideline [26, 27], which ensures rigorous methodology in both conduct and reporting. The comprehensive search protocol utilized major medical research databases, citation searching, and efforts to identify unpublished studies, which contributed to the inclusion of high-quality research. Notably, this is the first systematic review to investigate injury incidence in soccer referees, providing valuable insights and recommendations for future epidemiological studies. However, several limitations must be acknowledged. A significant limitation was the large heterogeneity among the included studies, which may stem from variations in injury measures, exposure times and definitions, and sample sizes. In this regard, injury distribution with respect to age, competition level, and history of prior injuries could not be analyzed due to limited and inconsistent data. In addition, variations among national leagues—such as differences in climate conditions, match congestion, competitive level, and geographical location—may have further complicated the interpretation of results. Notably, there was a marked discrepancy between retrospective and prospective studies: true prospective studies (*n* = 5) reported higher injury rates, while true retrospective studies (*n* = 7) reported significantly fewer. This indicates a clear recall bias [72] in retrospective studies, likely leading to an underestimation of the true injury incidence. Heterogeneity was also high in included papers, suggesting inconsistent study design and outcome measurements. This limited our ability to conduct interaction analysis. Exposure, particularly training time, was also poorly defined. Another important aspect highlighted by this review was the significant lack of epidemiological data on overuse injuries in soccer referees, including their prevalence and burden, representing a clear gap in the literature that deserves greater attention. Consequently, future research should emphasize the use of prospective designs with continuous and consistent injury recording to yield more accurate estimates.

## Conclusions

This systematic review and meta-analysis globally synthesize the available epidemiological literature on injuries in soccer referees. While making definitive overall conclusions remains challenging due to the heterogeneity of the included studies, important insights were gained regarding injury patterns across different referee cohorts. The review identified significantly higher injury rates during matches compared with training sessions, with OFRs experiencing more injuries than ARs. The most commonly affected anatomical regions were the lower extremities—particularly the thigh, knee, and calf—with sprains and strains being the predominant injury types. Importantly, this review highlights a critical gap in research on women’s soccer referees, underscoring the need for focused epidemiological studies in this population. To effectively reduce injury incidence and severity, future research should prioritize the development and evaluation of targeted preventive measures and training programs addressing the most frequent injuries, identified using repeatable and consistent measurements such as injury definition. Overall, the findings provide valuable epidemiological data and a foundation for researchers and practitioners worldwide to design and implement effective interventions aimed at enhancing the health and well-being of soccer referees.

## Supplementary Information

Below is the link to the electronic supplementary material.

Supplementary file1 (DOCX 18 KB)

Supplementary file2 (DOCX 141 KB)

Supplementary file3 (DOCX 22 KB)

Supplementary file4 (DOCX 25 KB)

Supplementary file5 (DOCX 25 KB)

## Declarations

### Availability of data and materials

All studies from which data were examined are cited in the references section.
