# Supplementary material for: Epidemiology of Injury in Elite and Amateur Soccer Referees: A Systematic Review and Meta-analysis
Source: Sports Med. 2025 Sep 29;55(12):3111–28. doi: 10.1007/s40279-025-02326-y (PMC12628465; doi:10.1007/s40279-025-02326-y)
Supplement: Supplementary file 1 — Supplementary file1 (DOCX 18 KB) [file 40279_2025_2326_MOESM1_ESM.docx]

**Supplementary File 1 (search strategy)**

**Title:** Epidemiology of injury in soccer referees: a systematic review and meta-analysis

**Search string:**

(soccer OR football) AND (injury OR wound OR incidence OR prevalence OR epidemiology) AND (referee)

| **Databases** | **Search query** |
| --- | --- |
| **Web of Science** | soccer OR football (Topic) AND injury OR wound OR incidence OR prevalence OR epidemiology (Topic) AND referee (Topic) |
| **PubMed** | ((soccer[Title/Abstract] OR football[Title/Abstract]) AND (injury[Title/Abstract] OR wound[Title/Abstract] OR incidence[Title/Abstract] OR prevalence[Title/Abstract] OR epidemiology[Title/Abstract])) AND (referee[Title/Abstract]) |
| **Medline** | ((soccer[Title/Abstract] OR football[Title/Abstract]) AND (injury[Title/Abstract] OR wound[Title/Abstract] OR incidence[Title/Abstract] OR prevalence[Title/Abstract] OR epidemiology[Title/Abstract])) AND (referee[Title/Abstract]) |
| **Scopus** | ( TITLE-ABS-KEY ( soccer OR football ) AND TITLE-ABS-KEY ( injury OR wound OR incidence OR prevalence OR epidemiology ) AND TITLE-ABS-KEY ( referee ) ) |
| **Cinahl** | ((soccer[Title/Abstract] OR football[Title/Abstract]) AND (injury[Title/Abstract] OR wound[Title/Abstract] OR incidence[Title/Abstract] OR prevalence[Title/Abstract] OR epidemiology[Title/Abstract])) AND (referee[Title/Abstract]) |
| **SPORTDiscus** | ((soccer[Title/Abstract] OR football[Title/Abstract]) AND (injury[Title/Abstract] OR wound[Title/Abstract] OR incidence[Title/Abstract] OR prevalence[Title/Abstract] OR epidemiology[Title/Abstract])) AND (referee[Title/Abstract]) |
