# Supplementary material for: Epidemiology of Injury in Elite and Amateur Soccer Referees: A Systematic Review and Meta-analysis
Source: Sports Med. 2025 Sep 29;55(12):3111–28. doi: 10.1007/s40279-025-02326-y (PMC12628465; doi:10.1007/s40279-025-02326-y)
Supplement: Supplementary file 2 — Supplementary file2 (DOCX 141 KB) [file 40279_2025_2326_MOESM2_ESM.docx]

**Leave-one-out Analysis**

| **Removed study** | **IRR Estimate** | **95% CI for IRR (lower)** | **95% CI for IRR (lower)** | **Tau-square (**$\boldsymbol{\tau}^{\boldsymbol{2}}$**)** | **I-square (**$\boldsymbol{I}^{\boldsymbol{2}}$**)** |
| --- | --- | --- | --- | --- | --- |
| Al Attar et al. | 1.427 | 0.943 | 2.158 | 2.431 | 98.497 |
| Bizzini et al. 1 | 1.359 | 0.911 | 2.027 | 2.268 | 98.41 |
| Bizzini et al. 1 | 1.451 | 0.961 | 2.192 | 2.411 | 98.482 |
| Bizzini et al. 1 | 1.41 | 0.933 | 2.131 | 2.423 | 98.488 |
| Bizzini et al. 1 | 1.383 | 0.919 | 2.082 | 2.371 | 98.403 |
| Bizzini et al.2 | 1.418 | 0.937 | 2.144 | 2.429 | 98.487 |
| Bizzini et al.2 | 1.439 | 0.952 | 2.176 | 2.424 | 98.505 |
| Bizzini et al.3 | 1.392 | 0.924 | 2.098 | 2.39 | 98.489 |
| Bizzini et al.3 | 1.425 | 0.943 | 2.154 | 2.425 | 98.513 |
| Bizzini et al.3 | 1.511 | 1.017 | 2.245 | 2.228 | 98.387 |
| Bizzini et al.3 | 1.477 | 0.983 | 2.219 | 2.356 | 98.468 |
| Bizzini et al.3 | 1.434 | 0.954 | 2.154 | 2.389 | 98.494 |
| Bizzini et al.3 | 1.436 | 0.956 | 2.159 | 2.39 | 98.495 |
| Bizzini et al.3 | 1.464 | 0.972 | 2.204 | 2.385 | 98.489 |
| Bizzini et al.3 | 1.518 | 1.023 | 2.253 | 2.207 | 98.367 |
| Bizzini et al.3 | 1.515 | 1.019 | 2.251 | 2.223 | 98.375 |
| Bizzini et al.3 | 1.52 | 1.025 | 2.255 | 2.199 | 98.358 |
| Bizzini et al. 4 | 1.367 | 0.914 | 2.044 | 2.302 | 98.435 |
| Bizzini et al. 4 | 1.38 | 0.919 | 2.072 | 2.352 | 98.468 |
| Bizzini et al. 4 | 1.5 | 1.004 | 2.241 | 2.282 | 98.407 |
| Bizzini et al. 4 | 1.48 | 0.986 | 2.224 | 2.348 | 98.437 |
| Bizzini et al. 4 | 1.447 | 0.959 | 2.183 | 2.41 | 98.505 |
| Bizzini et al. 4 | 1.447 | 0.958 | 2.184 | 2.413 | 98.506 |
| Da Silva et al. | 1.426 | 0.943 | 2.157 | 2.431 | 98.381 |
| Da Silva et al. | 1.444 | 0.956 | 2.183 | 2.421 | 98.444 |
| De Oliveiar et al. | 1.456 | 0.965 | 2.198 | 2.404 | 98.458 |
| De Oliveiar et al. | 1.427 | 0.943 | 2.158 | 2.431 | 98.386 |
| Gabrilo et al. | 1.394 | 0.924 | 2.103 | 2.398 | 98.455 |
| Gabrilo et al. | 1.397 | 0.926 | 2.109 | 2.405 | 98.451 |
| Gabrilo et al. | 1.392 | 0.923 | 2.1 | 2.394 | 98.469 |
| Gabrilo et al. | 1.392 | 0.923 | 2.099 | 2.394 | 98.467 |
| Kordi et al. | 1.354 | 0.909 | 2.016 | 2.245 | 98.384 |
| Kordi et al. | 1.4 | 0.927 | 2.114 | 2.41 | 98.477 |
| Kordi et al. | 1.375 | 0.916 | 2.064 | 2.341 | 98.452 |
| Kordi et al. | 1.395 | 0.925 | 2.105 | 2.401 | 98.459 |
| Matute-Llorente et al. | 1.43 | 0.946 | 2.162 | 2.429 | 98.506 |
| Matute-Llorente et al. | 1.446 | 0.958 | 2.184 | 2.416 | 98.504 |
| Matute-Llorente et al. | 1.4 | 0.927 | 2.113 | 2.409 | 98.466 |
| Matute-Llorente et al. | 1.426 | 0.943 | 2.157 | 2.43 | 98.505 |
| Matute-Llorente et al. | 1.402 | 0.928 | 2.118 | 2.413 | 98.473 |
| Matute-Llorente et al. | 1.46 | 0.969 | 2.201 | 2.393 | 98.493 |
| Matute-Llorente et al. | 1.467 | 0.975 | 2.208 | 2.377 | 98.484 |
| Matute-Llorente et al. | 1.45 | 0.961 | 2.189 | 2.411 | 98.502 |
| Matute-Llorente et al. | 1.467 | 0.975 | 2.208 | 2.377 | 98.484 |
| Matute-Llorente et al. | 1.45 | 0.961 | 2.189 | 2.411 | 98.502 |
| Moen et al. | 1.378 | 0.917 | 2.07 | 2.353 | 98.455 |
| Paes et al | 1.416 | 0.937 | 2.141 | 2.427 | 98.506 |
| Paes et al | 1.416 | 0.936 | 2.141 | 2.428 | 98.464 |
| Senisik et al. | 1.411 | 0.933 | 2.132 | 2.423 | 98.501 |
| Senisik et al. | 1.426 | 0.943 | 2.157 | 2.431 | 98.444 |
| Szymski et al. | 1.437 | 0.951 | 2.172 | 2.424 | 98.51 |
| Szymski et al. | 1.42 | 0.939 | 2.147 | 2.429 | 98.5 |
| Szymski et al. | 1.407 | 0.931 | 2.125 | 2.42 | 98.419 |
| Szymski et al. | 1.447 | 0.958 | 2.186 | 2.417 | 98.494 |
| Szymski et al. | 1.453 | 0.962 | 2.193 | 2.409 | 98.488 |
| Szymski et al. | 1.454 | 0.963 | 2.195 | 2.408 | 98.431 |
| Viera et al. | 1.403 | 0.929 | 2.119 | 2.414 | 98.474 |
| Viera et al. | 1.404 | 0.93 | 2.121 | 2.416 | 98.477 |
| Wilson et al. New | 1.383 | 0.919 | 2.081 | 2.37 | 98.467 |
| Wilson et al. New | 1.378 | 0.917 | 2.071 | 2.353 | 98.444 |

**Publication Bias Funnel Plots**


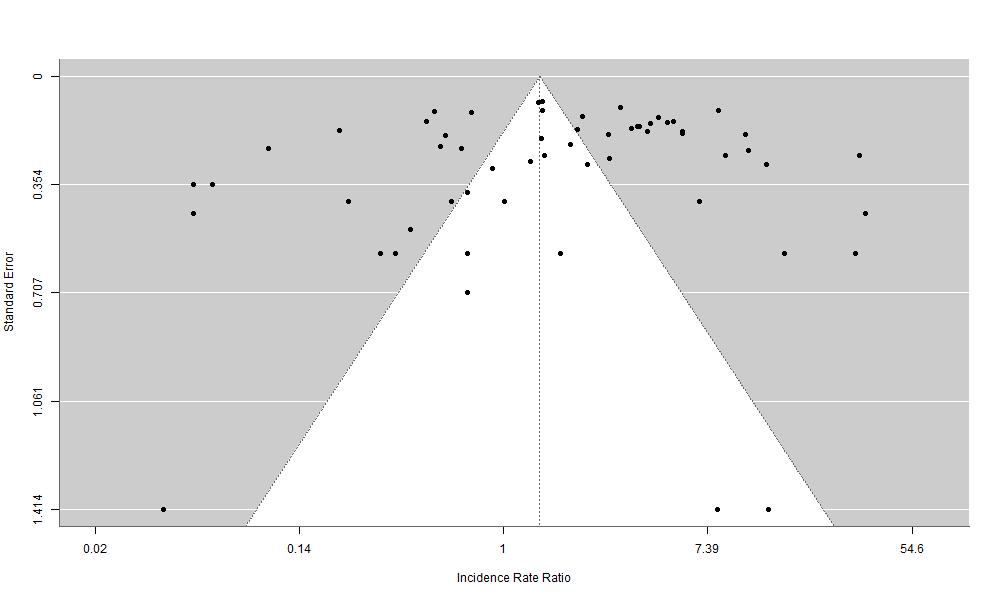


Funnel plot from original model


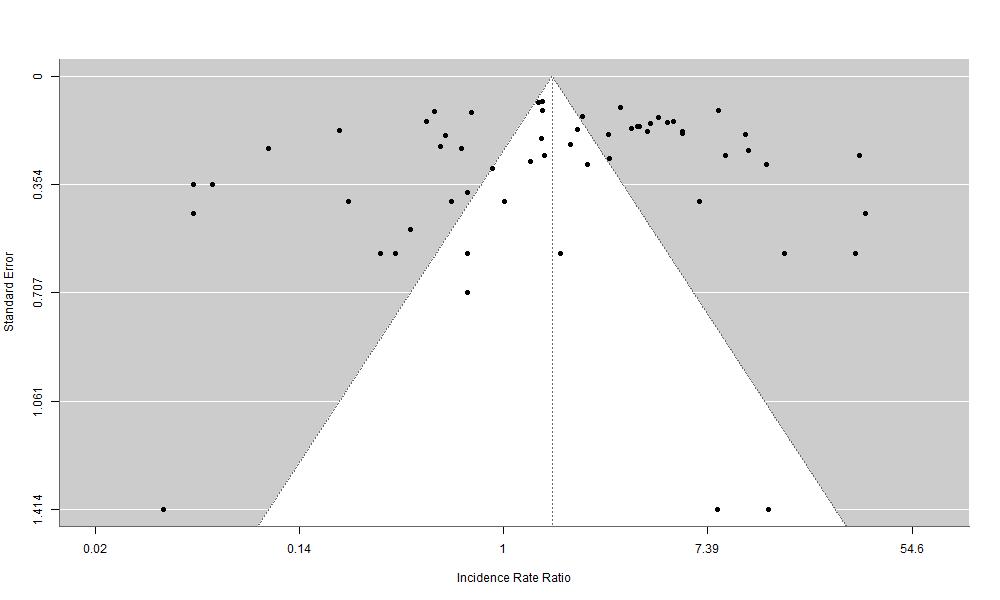


Funnel plot using mixed effect linear model on log incidence rate

**Outliers Analysis**


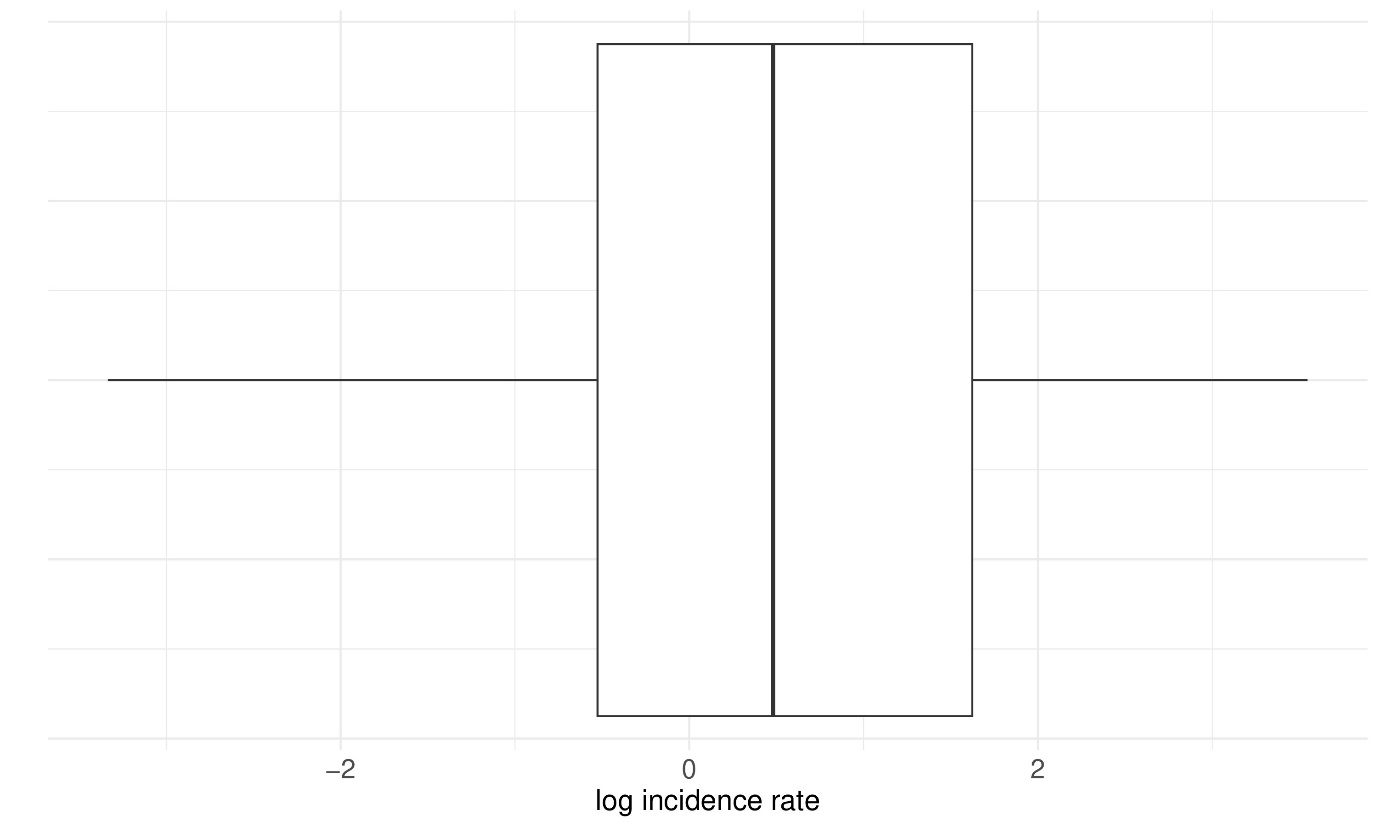


Box plot of log incidence rates
