# Supplementary material for: Epidemiology of Injury in Elite and Amateur Soccer Referees: A Systematic Review and Meta-analysis
Source: Sports Med. 2025 Sep 29;55(12):3111–28. doi: 10.1007/s40279-025-02326-y (PMC12628465; doi:10.1007/s40279-025-02326-y)
Supplement: Supplementary file 3 — Supplementary file3 (DOCX 22 KB) [file 40279_2025_2326_MOESM3_ESM.docx]

| **Reference** |  |  |  |  |  |  |  |  |  |  |  |  |  |  |
| --- | --- | --- | --- | --- | --- | --- | --- | --- | --- | --- | --- | --- | --- | --- |
|  | ***Selection*** | | | | | ***Comparability*** | | | ***Outcome*** | | | | ***Total***  ***Score*** | ***Rating*** |
|  | ***Q1*** | ***Q2*** | ***Q3*** | ***Q4*** | ***T*** | ***Q1a*** | ***Q1b*** | ***T*** | ***Q1*** | ***Q2*** | ***Q3*** | ***T*** |  |  |
| 1. Bizzini et al. (2009) | 🟌 | NA | 🟌 | 🟌 | 3 | 🟌 | 🟌 | 2 | 🟌 | 🟌 | 🟌 | 3 | **8** | **Low ROB** |
| 2. Bizzini et al. (2009) | 🟌 | NA | 🟌 | 🟌 | 3 | 🟌 | 🟌 | 2 |  | 🟌 | 🟌 | 2 | **7** | **Low ROB** |
| 3. Bizzini et al. (2009) | 🟌 | NA | 🟌 | 🟌 | 3 | 🟌 | 🟌 | 2 |  | 🟌 | 🟌 | 2 | **7** | **Low ROB** |
| 4. Bizzini et al. (2009) | 🟌 | NA | 🟌 | 🟌 | 3 | 🟌 | 🟌 | 2 | 🟌 | 🟌 | 🟌 | 3 | **8** | **Low ROB** |
| 5. Wilson et al. (2011) | 🟌 | NA | 🟌 | 🟌 | 3 | 🟌 | 🟌 | 2 |  | 🟌 | 🟌 | 2 | **7** | **Low ROB** |
| 6. Paes et al. (2011) | 🟌 | NA | 🟌 | 🟌 | 3 | 🟌 | 🟌 | 2 |  | 🟌 | 🟌 | 2 | **7** | **Low ROB** |
| 7. Gabrilo et al. (2013) | 🟌 | NA | 🟌 | 🟌 | 3 | 🟌 | 🟌 | 2 |  | 🟌 | 🟌 | 2 | **7** | **Low ROB** |
| 8. Kordi et al. (2013) | 🟌 | NA | 🟌 | 🟌 | 3 | 🟌 | 🟌 | 2 | 🟌 | 🟌 | 🟌 | 3 | **8** | **Low ROB** |
| 9. Da Silva et al. (2014) | 🟌 | NA | 🟌 | 🟌 | 3 | 🟌 | 🟌 | 2 |  | 🟌 | 🟌 | 2 | **7** | **Low ROB** |
| 10. De Oliveira et al. (2016) | 🟌 | NA | 🟌 | 🟌 | 3 | 🟌 | 🟌 | 2 |  | 🟌 | 🟌 | 2 | **7** | **Low ROB** |
| 11. Boneti Moreira et al. (2017) | 🟌 | NA | 🟌 | 🟌 | 3 | 🟌 | 🟌 | 2 |  | 🟌 | 🟌 | 2 | **7** | **Low ROB** |
| 12. Vieira et al. (2019) | 🟌 | NA | 🟌 | 🟌 | 3 | 🟌 | 🟌 | 2 |  | 🟌 | 🟌 | 2 | **7** | **Low ROB** |
| 13. Matute-Llorente et al. (2020) | 🟌 | NA | 🟌 | 🟌 | 3 | 🟌 | 🟌 | 2 | 🟌 | 🟌 | 🟌 | 3 | **8** | **Low ROB** |
| 14. Al Attar et al. (2021) ^†^ | 🟌 | NA | 🟌 | 🟌 | 3 | 🟌 | 🟌 | 2 | 🟌 | 🟌 | 🟌 | 3 | **8** | **Low ROB** |
| 15. Szymski et al. (2021) | 🟌 | NA | 🟌 | 🟌 | 3 | 🟌 | 🟌 | 2 |  | 🟌 | 🟌 | 2 | **7** | **Low ROB** |
| 16. Moen et al. (2022) | 🟌 | NA | 🟌 | 🟌 | 3 | 🟌 | 🟌 | 2 |  | 🟌 | 🟌 | 2 | **7** | **Low ROB** |
| 17. Senisik et al. (2022) | 🟌 | NA | 🟌 | 🟌 | 3 | 🟌 | 🟌 | 2 |  | 🟌 | 🟌 | 2 | **7** | **Low ROB** |
|  |  |  |  |  |  |  |  |  |  |  |  | **Mean** | **7.29** |  |
|  |  |  |  |  |  |  |  |  |  |  |  | **SD** | **0.45** |  |

**Descriptions**: Q: question, T: total score, 🟌: star awarded, NA: not applicable (item 2 cannot be assessed across all included studies, as none have a non-exposed group), ROB: risk of bias, †: This study was an randomized control trial, however, we only extracted the control group data and hence have judged this as a cohort study. Studies with NOS star scores of 0 to 4 were categorized as having a high ROB, those with scores of 5 to 6 as having a moderate ROB, and those with scores of 7 to 9 as having a low ROB.
